# Supplementary material for: Evaluation of soccer team defense based on prediction models of ball recovery and being attacked: A pilot study
Source: PLoS One. 2022 Jan 27;17(1):e0263051. doi: 10.1371/journal.pone.0263051 (PMC8794176; doi:10.1371/journal.pone.0263051)
Supplement: S1 Text — (PDF) [file pone.0263051.s002.pdf]

Supplementary Text 1

Here we general descriptions about 19 events in this study. Formally, the stuffs in the data company subjectively labeled each events.

1. Pass: a delivery the ball into one of their teammates
2. Cross: a delivery of the ball into the penalty area by the attacking team
3. Throw in: a play to throw the ball from behind a touchline to restart a play after the ball is kicked out by an opponent
4. Free kick: a kick opportunity given against the offensive team as a result of a foul outside the penalty area
5. Corner kick: a kick opportunity taken from the corner flag to restart a play when a player puts the ball behind their own goal line without a goal being scored
6. Trap: a play using their foot (or chest or thigh) to control the ball
7. Foul: a breach of the laws of the game by a player, followed by a free kick or penalty kick
8. Tackle: a play of winning the ball back from an opponent, achieved by using a leg
9. Interception: a play to prevent a pass from reaching its intended recipient
10. Shot: a play to get the ball past the goal line
11. Penalty kick: a kick taken a specific distance from goal, awarded e.g., when a team commits a foul inside its own penalty area
12. Own goal: scoring a goal against their own team as the result of an error
13. Goalkeeper hand clear: a goalkeeper's play to hand the ball away from the defending goal
14. Goalkeeper catch: a goalkeeper's play to catch the ball
15. Clearance: a play to kick the ball away from the defending goal
16. Block: a play that prevents the opponent team's shots by hitting the body
17. Dribble: running with the ball at their feet under control
18. Off-side: one of the laws of football relating to the positioning of defending players in relation to attacking players when the ball is played to an attacking player by a teammate
19. Goal kick: a kick to restart a play when the ball is played over the goal line by a player of the attacking team without a goal being scored
