# Supplementary figures and images for: Evaluation of soccer team defense based on prediction models of ball recovery and being attacked: A pilot study
Source: PLoS One. 2022 Jan 27;17(1):e0263051. doi: 10.1371/journal.pone.0263051 (PMC8794176; doi:10.1371/journal.pone.0263051)

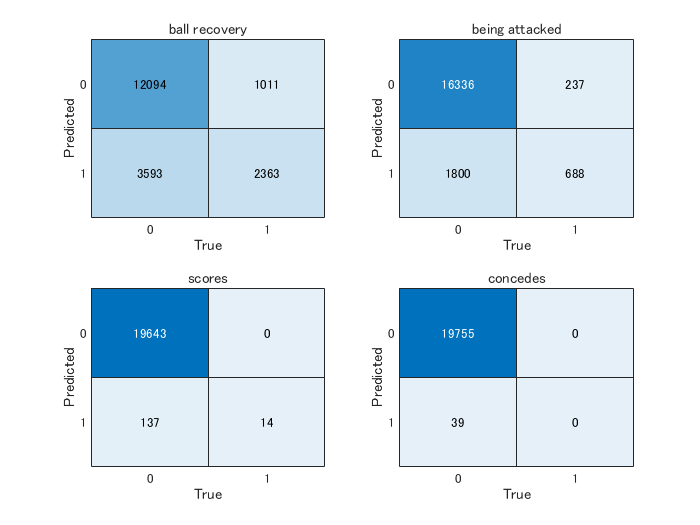

Supplement: S1 Fig — The numbers of actual and predicted pass recoveries in VDEP, being attacked in VDEP, scores in VAEP, and concedes in VAEP are shown. Note that these are the results of test data in the last 9 games (other games were used for training). (PNG) [file pone.0263051.s003.png]
